# High-Throughput Screening of COF Membranes and COF/Polymer MMMs for Helium Separation and Hydrogen Purification

**DOI:** 10.1021/acsami.2c04016

**Published:** 2022-04-28

**Authors:** Sena Aydin, Cigdem Altintas, Seda Keskin

**Affiliations:** † Department of Computational Science and Engineering, Koc University, Rumelifeneri Yolu, Sariyer, 34450 Istanbul, Turkey; ‡ Department of Chemical and Biological Engineering, Koc University, Rumelifeneri Yolu, Sariyer, 34450 Istanbul, Turkey

**Keywords:** COFs, membrane, gas separation, mixed matrix membranes, molecular simulations

## Abstract

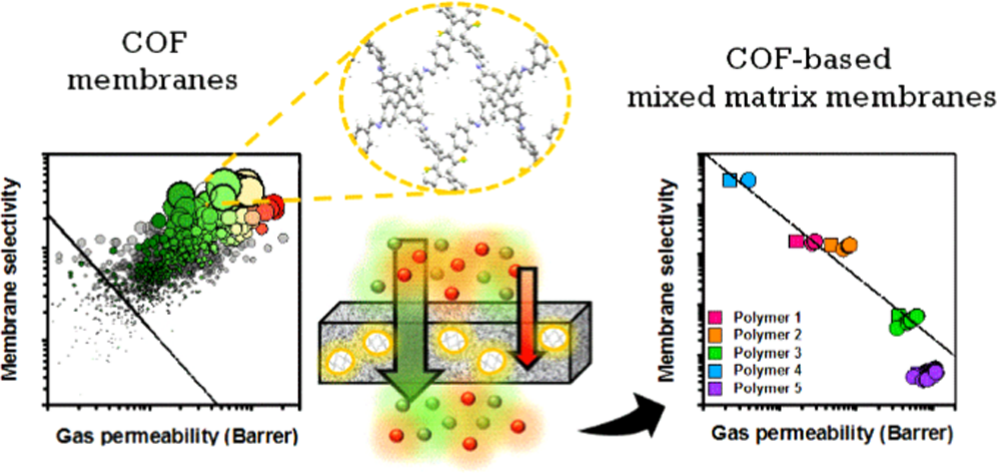

Hundreds of covalent organic frameworks (COFs) have been synthesized, and thousands of them have been computationally designed. However, it is impractical to experimentally test each material as a membrane for gas separations. In this work, we focused on the membrane-based gas separation performances of experimentally synthesized COFs and hypothetical COFs (hypoCOFs). Gas permeabilities of COFs were computed by combining the results of grand canonical Monte Carlo (GCMC) and molecular dynamics (MD) simulations, and many COF membranes were found to overcome the upper bound of polymeric membranes for He/H_2_, N_2_/CH_4_, H_2_/N_2_, He/CH_4_, H_2_/CH_4_, and He/N_2_ separations. We then examined the structure–permeability relations of the COF membranes that are above the upper bound for each of the six gas separations, and based on these relations, we proposed an efficient approach for the selection of the best hypoCOFs from a very large database. Molecular simulations showed that 120 hypoCOFs that we identified to be promising based on these structure–performance relations exceed the upper bound for He/CH_4_, He/N_2_, H_2_/CH_4_, and H_2_/N_2_ separations. Both real and hypothetical COFs were then studied as fillers in 25 different polymers, leading to a total of 29 020 COF/polymer and hypoCOF/polymer mixed matrix membranes (MMMs), representing the largest number of COF-based MMMs investigated to date. Permeabilities and selectivities of COF/polymer MMMs were computed for six different gas separations, and results revealed that 18 of the 25 polymers can be carried above the upper bound when COFs were used as fillers. The comprehensive analysis of COFs provided in this work will fully unlock the potential of COF membranes and COF/polymer MMMs for helium separation and hydrogen purification.

## 1 Introduction

Membrane-based gas separation, which utilizes the difference in solubility and diffusivity of different gases under a pressure gradient, is considered as an energy-efficient method since it does not require a phase change and can be achieved under relatively mild conditions.^1^ Industrial gas separations necessitate selective membranes and high gas permeabilities to reduce operating costs. Polymers have been the most widely studied membranes for various gas separations; however, they suffer from the trade-off between selectivity and permeability as defined by Robeson,^2,3^ which limits their performance in commercial processes. Due to this trade-off, mixed matrix membranes (MMMs) have been developed to combine the good processabilities of the polymers and outstanding gas separation performances of the porous filler materials such as zeolites,^4−6^ metal–organic frameworks (MOFs),^7−9^ and covalent organic frameworks (COFs).^10−14^

Due to the very large number and variety of MOFs, the number of computational screening studies on MOF membranes escalated quickly, while several studies focused on the identification of the most promising MOFs to be used in MMMs for gas separations.^15−18^ For example, gas permeabilities of 13 MOFs computed using molecular simulations were utilized to predict H_2_/N_2_ separation performances of 78 MOF-based MMMs. Several MMMs were shown to exceed the upper bound due to almost 2 times higher H_2_ permeabilities than those of the pure polymers.^17^ H_2_/CH_4_ separation performances of 119 MOF-based MMMs consisting of 17 different MOFs and 7 polymers were investigated using molecular simulations,^18^ and it was shown that MOF-based MMMs could increase both the H_2_ permeability and H_2_/CH_4_ selectivity of polymers.

COFs, porous crystalline materials consisting of covalently bonded light elements including boron, silicon, carbon, nitrogen, and oxygen, have been recently discovered and shown to have a high potential for gas separations due to their chemically diverse and robust structures.^19^ COFs exhibit good compatibility with the polymer matrix thanks to their organic parts. Therefore, in addition to the common advantages of MOFs such as structural tunability and high accessible surface area, the affinity and miscibility of COFs to polymers provide an additional benefit to using them as fillers in MMMs.^20^ COF-based MMMs have recently been investigated experimentally for the separation of several gas pairs.^14,20−29^ Addition of a COF filler, NUS-2, into the polybenzimidazole (PBI) membrane significantly increased the H_2_/CO_2_ selectivity of PBI from 9.5 to 31.4 at 5 bar and 308 K and carried the polymer over the upper bound.^10^ Incorporation of a Schiff base network-type COF (SNW-I) into a polyimide (PI) membrane increased the CO_2_ permeability of the membrane from 6 to 12.4 Barrer and the CO_2_/CH_4_ selectivity from 9 to 13.4 at 4 bar and 298 K.^30^ COF nanosheet clusters (TpPa-1-nc) increased the selectivity of polyether-block-amide (PEBA) membranes from ∼42 to 72 and the CO_2_ permeability from ∼4.5 to 7.5 Barrer at 3 bar and 298 K for the separation of an equimolar CO_2_/N_2_ mixture.^14^ A 2D-COF-based MMM synthesized using cross-linked poly(ethylene oxide) (XLPEO) exceeded Robeson’s upper bound with its high CO_2_ permeability (804 Barrer), and CO_2_/N_2_, CO_2_/CH_4_, and CO_2_/H_2_ selectivities of 61, 20, and 15, respectively, at 5 bar and 308 K, which were higher than the CO_2_ permeability (432 Barrer) and CO_2_ selectivities (35, 14, and 11, respectively) of the pure XLPEO membrane.^13^

The insight gained from the computational screening of COFs for membrane applications could be of substantial importance for deciding on the next COF filler to be used in experimental MMM studies. Recent computational studies predicted gas separation properties of COF membranes.^31−37^ For example, Yang et al.^31^ demonstrated that COF-102, -103, -105, and -108 have higher selectivities (11.7, 14, 28.8, and 33.2, respectively) compared to IRMOF-1 and Cu-BTC for membrane-based H_2_/CH_4_ separation at 10 bar and 298 K. 295 COF membranes were investigated for CO_2_/N_2_ separation at 1 bar and 10 bar, at 298 K, and results showed that CO_2_ permeabilities of COF membranes overcome those of MOF membranes, while MOF membranes have higher CO_2_/N_2_ selectivities than COF membranes.^34^ A computational investigation on the H_2_/CO_2_ separation performances of 288 COF membranes at 10 bar and 298 K has shown that COF membranes significantly outperform polymeric membranes due to their H_2_ permeabilities ranging from 599 to 1.5 × 10^6^ Barrer and H_2_/CO_2_ selectivities of up to 4.74.^35^ H_2_/CO_2_ separation performances of 794 hypoCOF membranes were shown to be even superior, with H_2_ permeabilities in the range of 9 × 10^5^–5 × 10^6^ Barrer and H_2_/CO_2_ selectivities between 2.66 and 6.14.^36^ A total of 572 COFs were studied for membrane-based H_2_/CH_4_ separation at 1 bar and 298 K, and several COFs with large porosities showed a good combination of H_2_ permeability (>10^5^ Barrer) and H_2_/CH_4_ selectivities up to 4.6.^37^ In contrast to COF membranes, a very limited number of studies, only two, focused on computational modeling of COF/polymer MMMs. Twenty-nine COFs were computationally evaluated as fillers in three polymers for CO_2_/CH_4_ separation, and several MMMs were found to be above the upper bound due to the increase in their CO_2_ permeability up to ∼1900 Barrer and CO_2_/CH_4_ selectivity up to ∼45.^38^ Gulbalkan et al.^39^ investigated the N_2_/CH_4_ separation performances of 25 different COF/polymer MMMs and showed that MMMs with improved N_2_ permeabilities (∼4.5 to 10^4^ Barrer) could be obtained upon the incorporation of COFs into polymers.

Since the number of experimentally synthesized COFs has already exceeded 600,^40^ in addition to the existence of hundreds of polymers, experimental investigation of every possible COF/polymer MMM is very challenging. The number of experimental and computational studies on evaluating gas separation performances of COF-based MMMs is very limited, as demonstrated above, and these MMMs have been mostly tested for CO_2_ separation. In addition to these, we still do not know the potential of the hypothetical, computer-generated COFs (hypoCOFs)^41−43^ as fillers in MMMs although they may have similar or even better gas separation performances than the experimentally available COFs. Motivated by these, we aimed to reveal the potential of all synthesized and computer-generated COFs as fillers in MMMs for a variety of gas separations. We first focused on the most recent COF database consisting of 648 experimentally synthesized structures. He, H_2_, CH_4_, and N_2_ permeabilities of these COFs were predicted under realistic conditions (1 bar, 298 K) by performing grand canonical Monte Carlo (GCMC) and molecular dynamics (MD) simulations. Performances of 589 COF membranes and 24 100 COF/polymer MMMs composed of 25 different polymers were examined for six different gas separations, He/H_2_, He/CH_4_, He/N_2_, H_2_/CH_4_, H_2_/N_2_, and N_2_/CH_4_, which represent both the largest number of COF-based membranes and the largest variety of gas separations studied with COFs to date. Structural properties of the experimentally synthesized COF membranes, which were identified to be above Robeson’s upper bound, were determined, and the obtained results were used to select 120 representative hypoCOFs from a very large material database. Membrane-based gas separation performances of these hypoCOFs were predicted by conducting GCMC and MD simulations and compared with polymer, COF, and MOF membranes. Finally, gas permeabilities and selectivities of 4920 hypoCOF-incorporated MMMs were computed for all six gas separations. Overall, we revealed the gas permeability and selectivity of a total of 709 COF and hypoCOF membranes and 29 020 COF/polymer and hypoCOF/polymer MMMs for six gas separations. The results presented in this work will significantly accelerate the design of COF-based MMMs by directing the experimental efforts and sources to the development of the most promising COF materials.

## 2 Computational Details

**Scheme 1:**
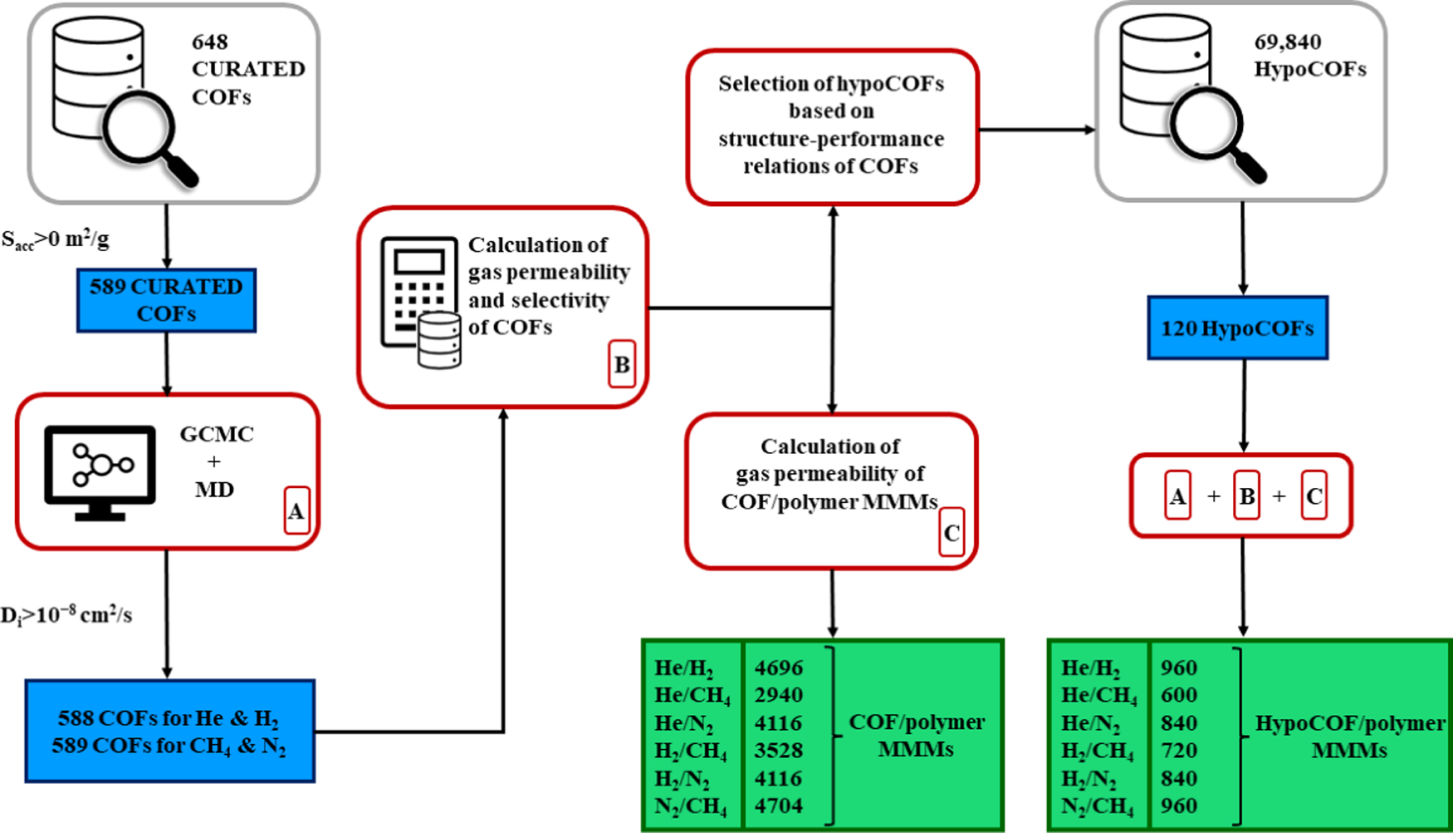
Computational Methodology Used in This Study

A high-throughput computational screening approach, as shown in Scheme 1, was proposed and used in this work to compute the gas permeabilities and selectivities of COF membranes and COF/polymer MMMs for He/H_2_, He/N_2_, He/CH_4_, H_2_/N_2_, H_2_/CH_4_, and N_2_/CH_4_ separations. We worked with 648 Clean, Uniform, and Refined with Automatic Tracking from Experimental Database (CURATED) COF structures.^40^ All of the structural properties of COFs that we used in this work, the largest cavity diameter (LCD), the pore limiting diameter (PLD), the accessible surface area (*S*_acc_), density (ρ), and porosity (ϕ), were computed using Zeo++ software.^44^ The widely applied experimental method for measuring the surface area of porous materials is the Brunauer–Emmett–Teller (BET), which is based on nitrogen adsorption in porous materials.^45^ Therefore, we utilized a probe radius of 1.86 Å (representing the radius of the nitrogen molecule) to predict the accessible surface area of materials investigated in this work, and the number of COFs was refined as 589 to only study the COFs with *S*_acc_ > 0 m^2^/g as membranes.

First, we performed grand canonical Monte Carlo (GCMC) simulations to compute He, H_2_, N_2_, and CH_4_ uptakes in COFs (*N*_i_^COF^) at 1 bar and 298 K. Molecular dynamics (MD) simulations were performed to calculate the self-diffusivity of the gas i in the COF (*D*_i_^COF^) at the loadings obtained from GCMC simulations. RASPA software (version 2.0.35) was used for all molecular simulations in this work.^46^ He,^47^ H_2_,^48^ and CH_4_^47^ were modeled as nonpolar and single-site spheres, while N_2_^49^ was represented with a three-site model consisting of two N atoms and a dummy atom as the center-of-mass. He and CH_4_ were described with TraPPE models.^47,50^ The parameters for H_2_ were acquired from the Buch potential,^48^ and the parameters from the work of Makrodimitris et al.^49,51^ were used for N_2_. Intermolecular interactions between host–gas and gas–gas were defined using the Lennard–Jones 12-6 (LJ) potential. The interaction parameters for COF atoms were obtained from the universal force field (UFF).^52^ The cutoff distance was set to 14 Å to truncate the LJ interactions, and Lorentz–Berthelot mixing rules were applied. The Coulomb potential was used to compute the electrostatic interactions between N_2_ and COF atoms. Long-range electrostatic interactions were considered using the Ewald summation.^53^ Density-derived electrostatic and chemical (DDEC) charges were already assigned to the CURATED COFs in the database,^40^ and the charge equilibration method (Qeq)^54^ was used to assign the partial charges to hypothetical COFs that we studied. In GCMC simulations, 10 000 cycles were performed for initialization, and 20 000 cycles were used for taking the ensemble averages for gas uptakes.

MD simulations were carried out in the canonical ensemble (NVT) up to 5 ns with a time step of 1 fs to compute the *D*_i_^COF^ values at 298 K using the Nosé–Hoover thermostat.^55^ Self-diffusion coefficients of gases in COFs were calculated using the slope of the mean square displacements according to Einstein’s relation.^56^ After carefully examining the results of MD simulations, we considered only the COFs with *D*_i_^COF^ > 10^–8^ cm^2^/s for the accurate characterization of molecular diffusion and ended up with 588 COFs for He and H_2_ and 589 COFs for N_2_ and CH_4_. The gas uptakes and self-diffusivities were used to calculate the single-component gas permeability of COFs (*P*_i_^MMM^) as shown with (a) in Table 1. Here, *c*_i_, *D*_i_, and *f*_i_ are the uptake, self-diffusivity of gas i, and feed side pressure of the membrane, respectively, and the permeate was assumed to be at vacuum.^57^ Using the ratio of gas permeabilities, ideal membrane selectivities of COFs (*S*_i/j_^COF^) were calculated using (b) in Table 1.

**Table 1:** Calculation of the Gas Permeability and Selectivity of Membranes

COF membranes		COF/polymer MMMs	
(a)	*P*_i_^COF^ = *c*_i_^COF^ × *D*_i_^COF^/*f*_i_	(c)	
(b)	*S*_i/j_^COF^ = *P*_i_^COF^/*P*_j_^COF^	(d)	*S*_i/j_^MMM^ = *P*_i_^MMM^/*P*_j_^MMM^

We aimed to investigate the performances of COF-based MMMs consisting of different types of polymers and COF fillers for He/H_2_, He/N_2_, He/CH_4_, H_2_/N_2_, H_2_/CH_4_, and N_2_/CH_4_ separations. A total of 25 polymers establishing Robeson’s upper bound for each gas separation were chosen to generate COF/polymer MMMs. These polymers are enlisted in Tables S1–S6 together with their experimentally reported gas permeabilities that we collected from the literature. Various theoretical permeation models were used in the literature^58,59^ to predict gas permeabilities of MMMs. The Maxwell model, the most widely used permeation model to predict the gas separation performances of MMMs, especially at low filler loadings (volume fraction ≤ 0.2), was used in this work, as shown in expression (c) in Table 1. Here, φ is the volume fraction of COFs used as fillers in the polymer matrix, *P*_i_^P^ represents the gas permeability of the neat polymer taken from the literature, *P*_i_^COF^ is the gas permeability that we calculated using molecular simulations, and *P*_i_^MMM^ is the gas permeability of COF/polymer MMM. We used the filler loading (φ) as 0.2 to be in the range of applicability of the Maxwell model.^60^ Selectivities of MMMs (*S*_i/j_^MMM^) were calculated via expression (d) in Table 1.

In the last part of this work, we aimed to examine the gas separation performances of hypoCOFs selected from a very large data set of 69 840 computationally designed structures.^61^ Since performing molecular simulations, especially MD, for this very large material space is computationally challenging, we introduced an efficient approach for the targeted selection of the most promising hypoCOF membrane candidates. First, we focused on the physical features (PLD, LCD, ϕ, and *S*_acc_) of the promising CURATED COFs, which were shown to be above Robeson’s upper bound because of their high gas permeabilities (>4 × 10^5^ Barrer) based on the results of our molecular simulations. We defined and considered the ranges of structural properties of the promising COF membranes, 20 Å < LCD < 57 Å, 17 Å < PLD < 57 Å, 0.77 < ϕ < 0.95, 2000 < *S*_acc_< 10 000 m^2^/g, and ρ > 0.1 g/cm^3^, to narrow down the set of hypoCOFs. We then divided the set of hypoCOFs into two parts: (i) 2 dimensional (2D) hypoCOFs and (ii) 3D-hypoCOFs. *S*_acc_ and ϕ are the physical features having the most significant effect on the gas permeabilities of COFs, as we will discuss later, and by considering them, we created four mini-hypoCOF sets composed of 2D and 3D structures: hypoCOFs_2D-Sacc_, hypoCOFs_3D-Sacc_, hypoCOFs_2D-ϕ_, and hypoCOFs_3D-ϕ_. In each set, 30 hypoCOFs exist, based on the highest, mean, and the lowest *S*_acc_ and ϕ.

## 3 Results and Discussion

### 3.1 COF Membranes

**Figure 1:**
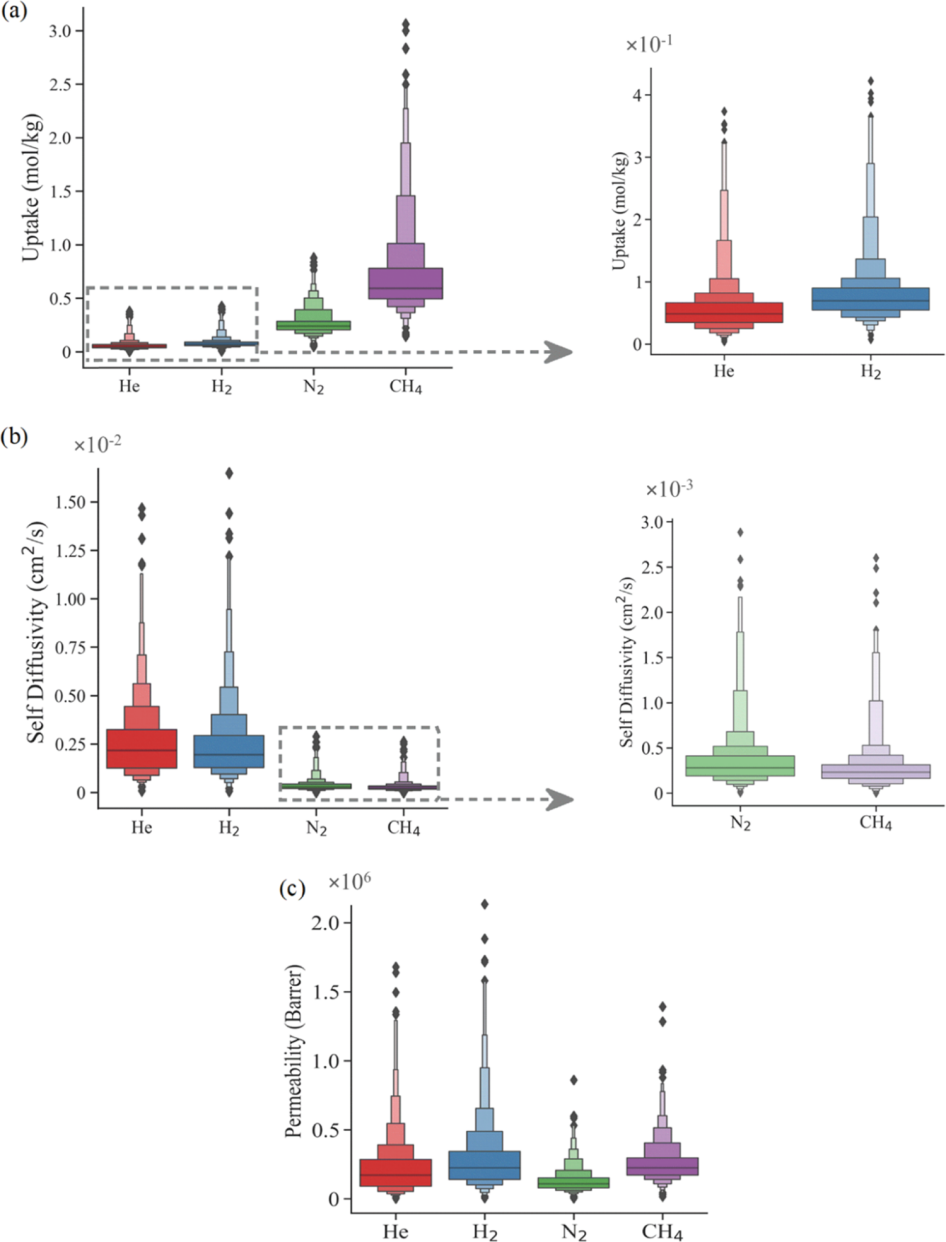
Ranges of (a) uptakes, (b) self-diffusion coefficients, and (c) permeabilities of He, H_2_, N_2_, and CH_4_ in 589 CURATED COFs computed at 1 bar and 298 K. Heights (widths) of the boxes represent certain numeric ranges for a property (the relative distribution of materials among all materials with that specific property in the given numeric range).

Uptakes and self-diffusivities for He, H_2_, N_2_, and CH_4_ gases in COFs and the gas permeabilities of COF membranes are shown in Figure 1. Figure 1a illustrates the range of uptakes for each gas in COFs. He and H_2_ have the lowest uptakes (in the ranges of 6.45 × 10^–3^–0.37 and 7.42 × 10^–3^–0.42 mol/kg, respectively) in COFs as a result of their low interaction energies with the COF atoms.^62^ The relatively higher CH_4_ uptakes compared to other gases is due to the stronger interaction of CH_4_ (ε/*k*_B_ value for LJ interactions is 148 K) with the frameworks compared to N_2_, H_2_, and He (ε/*k*_B_ values for LJ interactions are 38.3, 34.2, and 10.9 K for N_2_, H_2_, and He, respectively).

Figure 1b demonstrates the range of self-diffusivity of He, H_2_, CH_4_, and N_2_ in COFs. Diffusion coefficients are inversely proportional to the adsorption properties of gases since a strongly adsorbed gas molecule within COFs cannot diffuse easily, and vice versa. CH_4_ and N_2_ molecules are heavier than He and H_2_; hence, self-diffusivities of He and H_2_ are relatively higher (7.68 × 10^–5^–1.47 × 10^–2^ and 5.88 × 10^–5^–1.65 × 10^–2^ cm^2^/s, respectively) than those of CH_4_ and N_2_. This is in agreement with the previously reported self-diffusion coefficients of the same gases in MOFs for which He and H_2_ diffusions were found to be faster compared to other gas molecules (1.36 × 10^–4^–1.87 × 10^–2^ cm^2^/s for He and 1.24 × 10^–5^–1.31 × 10^–2^ cm^2^/s for H_2_).^63^

As shown in Figure 1c, the gas permeabilities of COFs, *P*_He_, *P*_H_2__, *P*_N_2__, and *P*_CH_4__, were computed to be in the ranges of 2.84 × 10^3^–1.68 × 10^6^, 5.59 × 10^3^–2.14 × 10^6^, 4.11 × 10^3^–8.61 × 10^5^, and 1.58 × 10^4^–1.39 × 10^6^ Barrer, respectively. The lower and upper limits of permeability for each gas are similar due to the inverse relationship between adsorbed loading and self-diffusion. The impact of diffusion properties on determining the gas permeability seems more dominant for He and H_2_, as shown in Figure 1c. Self-diffusion coefficients of CH_4_ are the lowest, but CH_4_ permeabilities of COFs are high, on the order of 10^4^–10^6^ Barrer, since CH_4_ uptakes vary in a wider range compared to the uptakes of He, H_2_, and N_2_. This indicates that the impact of adsorption on CH_4_ permeability of COFs is more pronounced than the impact of diffusion.

**Figure 2:**
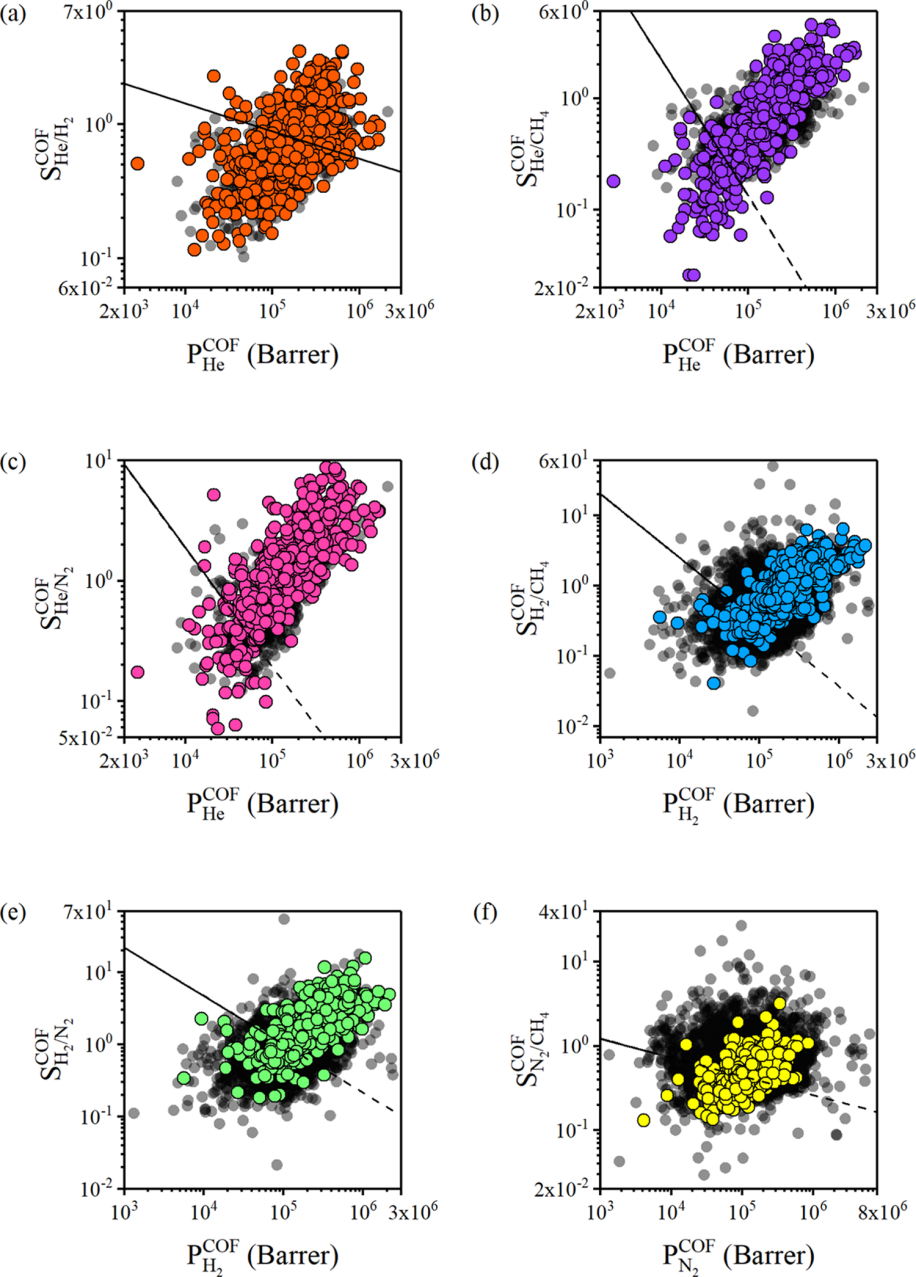
Permeabilities and selectivities of CURATED COFs for (a) He/H_2_, (b) He/CH_4_, (c) He/N_2_, (d) H_2_/CH_4_, (e) H_2_/N_2_, and (f) N_2_/CH_4_ separations. Colored points represent the data of COFs computed in this work, whereas black points show the data of MOFs that we computed in a previous work under the same conditions.^63^ Robeson’s upper bound is shown with the black line.

We computed the permeabilities and selectivities of 587 COF membranes for He/H_2_, 588 COF membranes for He/CH_4_, He/N_2_, H_2_/CH_4_, and H_2_/N_2_, and 589 COF membranes for N_2_/CH_4_ separation at 1 bar and 298 K, as shown in Figure 2. Gas separation performances of 5599 MOF membranes computed in our previous work^63^ were also included to comprehensively compare COFs and MOFs under the same conditions. We first discuss He/H_2_, He/CH_4_, and He/N_2_ separations as illustrated in Figure 2a–c, respectively, since He is a valuable noble gas for which the area of utilization ranges from medical to industrial applications,^64,65^ and it has to be purified from H_2_, CH_4_, and N_2_.

Figure 2a depicts simulated He permeabilities and He/H_2_ selectivities of 587 COFs (orange data points) and 864 MOFs (black data points). He permeabilities of COFs were computed to be in the range of 2.84 × 10^3^–1.68 × 10^6^ Barrer with He/H_2_ selectivities in the range of 0.12–3.5. Selectivity values close to 1 indicate that permeabilities of He and H_2_ are similar due to their weak interactions with the framework atoms and lower molecular weights, causing similar uptakes and diffusivities as was previously shown in Figure 1c. Although the highest and the lowest values for He permeability of MOFs (8.06 × 10^3^–2.09 × 10^6^ Barrer) have the same orders as those of COFs, selectivities of MOFs (0.1–2.3) are lower than COFs. Therefore, it can be interpreted that COFs could be more useful membranes than MOFs for this separation.

He permeabilities and He/CH_4_ selectivities of 588 COFs (purple data points) and 864 MOFs (black data points) are shown in Figure 2b. We extended the upper bound with a dashed line since COFs and MOFs have comparatively higher gas permeabilities than the experimentally reported permeabilities of polymers. For He/CH_4_ separation, the selectivities of COFs and MOFs are similar, between 2.6 × 10^–2^–4.5 and 2 × 10^–2^–4.2, respectively. Although the selectivities for He/CH_4_ separation reach up to 4.5 in COFs and MOFs, there are strongly CH_4_-selective materials in both groups. Besides, in contrast to the trade-off observed in polymeric membranes, the correlation between permeability and selectivity follows a linear trend for both COFs and MOFs. There are 499 COFs among 588 COFs that could overcome the upper bound for He/CH_4_ separation, as shown in Figure 2b, showing the promise of COFs for this gas separation. He permeabilities and He/N_2_ selectivities of 588 COFs (pink data points) are shown in Figure 2c. For He/N_2_ separation, the selectivity varies between 5.8 × 10^–2^–8.7 and 0.12–6.1 for COFs and MOFs, respectively. COFs have a linear correlation between He permeability and selectivity, and 539 COFs among 588 COFs were found to be above the upper bound for He/N_2_ separation, indicating their high potential for this gas separation.

Hydrogen is one of the energy carriers in great demand since it covers expectations as a clean, less-carbon-emitting alternative with high efficiency to overcome environmental pollution. Figure 2d,e shows the performances of 588 COF membranes for H_2_/CH_4_ and H_2_/N_2_ separations, respectively. COFs have a strong correlation between permeability and selectivity for H_2_/CH_4_ and H_2_/N_2_ separations in contrast to the trade-off observed in polymeric membranes. Calculated H_2_ permeabilities of COFs vary between 5.59 × 10^3^ and 2.14 × 10^6^ Barrer for H_2_/CH_4_ separation, as shown in Figure 2d. MOFs comprise a broader H_2_ permeability range; hence, there are MOFs with lower or higher H_2_ permeabilities compared to COFs. H_2_/CH_4_ selectivities of MOFs were reported to be up to 1 order higher (1.7 × 10^–2^–50.1) compared to COFs, whereas selectivities of COFs change between 4.1 × 10^–2^ and 6.3. This can be attributed to the presence of narrower pores in MOFs, which have stronger interactions with CH_4_ molecules compared to COFs.^66^ A total of 416 and 535 COFs among 588 exceed the upper bound for H_2_/N_2_ and H_2_/CH_4_ separations, respectively.

Membrane-based technologies are preferred as they are less costly and more energy-efficient compared to cryogenic distillation to separate N_2_ from CH_4_ for the purification of natural gas. We studied N_2_ permeabilities and N_2_/CH_4_ selectivities of 589 COFs (yellow data points) and compared them with 5599 MOFs (black data points) in Figure 2f. Calculated N_2_ permeabilities and N_2_/CH_4_ selectivities of COFs were in the range of 4.11 × 10^3^–8.61 × 10^5^ Barrer and 0.13–3.2, respectively. The low N_2_ selectivity of COFs can be explained by their large pore sizes, which let both N_2_ and CH_4_ pass through.

**Figure 3:**
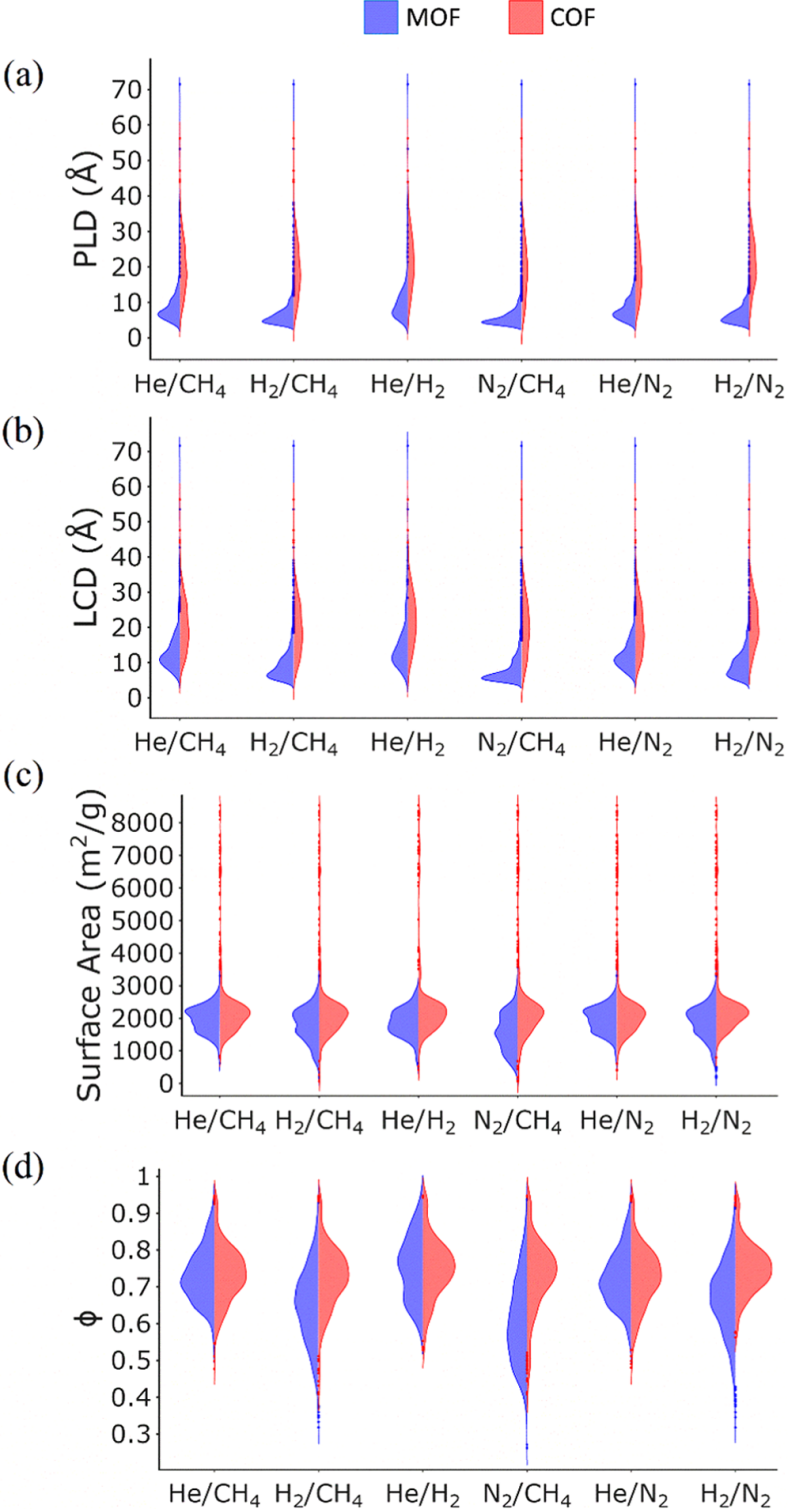
Distribution of (a) PLD, (b) LCD, (c) *S*_acc_, and (d) ϕ of COFs and MOFs, which are above the upper bound for each gas separation. Data for MOFs are taken from our previous study.^63^

After identifying the COFs that surpass the upper bound for each gas separation, we examined the PLD, LCD, ϕ, and *S*_acc_ of these materials (all sets of materials) in Figure 3 (Figure S1). Among the 589 COFs that we examined in this study, 262, 499, 539, 535, 416, and 347 COFs were found to be above the upper bound for He/H_2_, He/CH_4_, He/N_2_, H_2_/CH_4_, H_2_/N_2_, and N_2_/CH_4_ separations, respectively. For all separations, PLD and LCD of COFs, which are above the upper bound (shown in red in Figure 3a,b), vary in a broad range. In contrast, for the MOFs that were previously identified to be above the upper bound (shown in blue in Figure 3), PLD and LCD vary in narrow ranges.^63^ The medians of PLD (between 5 and 8.9 Å) and LCD (between 6.7 and 12.6 Å) of MOFs were found to be smaller than those of COFs (for PLD between 16.4 and 21.4 Å, for LCD between 16.9 and 22.3 Å), since the gas permeability of some MOFs has a stronger dependency on the pore size compared to that of COFs. We provided the properties of the best-performing MOF and COF membrane candidates, 10 materials that are located above the upper bound with the highest selectivities and permeabilities for each separation, in an Excel document as a part of the Supporting Information. Permeability is affected by adsorption and diffusion of gas molecules, which involve a complex interplay of several parameters of structures such as pore size, surface area, porosity, and chemical environment. Most of the COFs naturally have larger pores than many MOFs, and as an outcome, the PLD and LCD of COFs above the upper bound vary over a wide range compared to MOFs. Figure 3c shows that *S*_acc_ of COFs and MOFs vary in a very distinct range. For COFs, the range of *S*_acc_ is 792.6–8533.8 m^2^/g, and MOFs exhibit a wider distribution with smaller surface areas compared to COFs. Figure 3d illustrates that MOFs and COFs, which are above the upper bound, exhibit similar ranges of ϕ for He/CH_4_, He/N_2_, and He/H_2_ separations. Overall, Figure 3 suggests that surface area and porosity can be interpreted as important features to select the COF membranes located above the upper bound.

After showing the high potential of CURATED COFs for gas separations, we studied hypoCOFs as membranes. Since it is not practical to perform the computationally demanding MD simulations for all 69 840 hypoCOFs available in the database, we proposed an approach for the targeted selection of hypoCOFs. We examined the structure–performance relations of CURATED COFs and identified the structural properties of the most promising COF membranes. We then selected hypoCOFs based on these structural properties. The relations between PLD, LCD, *S*_acc,_ and ϕ of COFs and their H_2_, CH_4_, He, and N_2_ permeabilities are demonstrated in Figure S2. A total of 142 common COFs (colored data points) out of 589 COFs (gray data points) surpassed the upper bound for all six gas separations that we considered in this work, with high permeability (>4 × 10^5^ Barrer for the strongly adsorbed gas component). These 142 COFs have PLD, LCD, *S*_acc_, and ϕ ranges of 17–57 Å, 20–57 Å, 2000–10 000 m^2^/g, and 0.77–0.95, respectively, and have ρ > 0.1 g/cm^3^. When the set of hypoCOFs was refined according to these specified ranges, 23 341 hypoCOFs remained, and we grouped them as 2D and 3D. Finally, based on Figure 3, which showed the importance of *S*_acc_ and ϕ to achieve high gas permeability, we created four representative hypoCOF sets, each consisting of 30 hypoCOFs having either 2D or 3D structures, with the highest, mean, and the lowest *S*_acc_ or ϕ, and named as hypoCOFs_2D-Sacc_, hypoCOFs_3D-Sacc_, hypoCOFs_2D-ϕ_, and hypoCOFs_3D-ϕ_, respectively.

**Figure 4:**
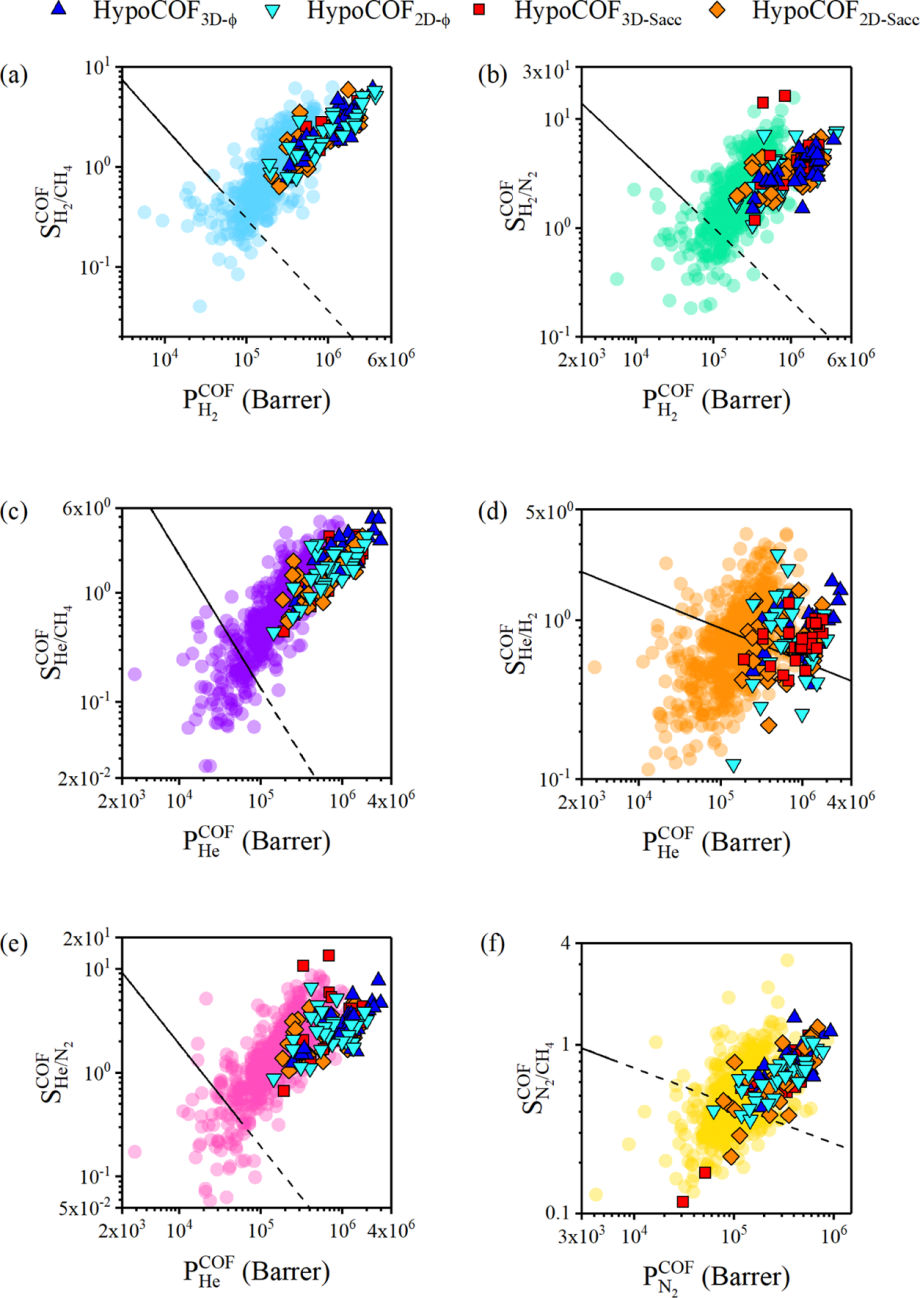
Comparison of CURATED COFs and selected hypoCOFs in terms of gas permeability and membrane selectivity for (a) H_2_/CH_4_, (b) H_2_/N_2_, (c) He/CH_4_, (d) He/H_2_, (e) He/N_2_, and (f) N_2_/CH_4_ separations. The data points (circles) in the background show the results of CURATED COFs obtained in this work.

We computed the gas permeability and selectivity of 120 hypoCOF membranes and showed them in Figure 4 together with the results of 589 CURATED COF membranes. For H_2_/CH_4_, H_2_/N_2_, He/CH_4_, and He/N_2_ separations, all hypoCOFs overcome the upper bound with their high gas permeabilities and selectivities as shown in Figure 4a,b,c,e. Only for He/H_2_ and N_2_/CH_4_ separations (Figure 4d,f), several hypoCOFs are located below the upper bound. HypoCOFs have almost the same He and H_2_ permeabilities due to their weak interactions with the frameworks. Thus, slightly H_2_/He-selective hypoCOFs could not surpass the upper bound. For N_2_/CH_4_ separation, N_2_ permeabilities of hypoCOFs vary in a broader range compared to CH_4_ permeabilities due to the high diffusivity of N_2_. Eight hypoCOF membranes below the upper bound have low N_2_ diffusivities, and therefore low permeabilities, which make these hypoCOFs CH_4_ selective with CH_4_ permeabilities in the range of 1.28 × 10^5^–9.60 × 10^5^ Barrer. Overall, these results show that our approach for the targeted selection of hypoCOFs based on the structure–performance relations of CURATED COFs accurately identifies the most promising hypoCOF candidates among several thousands. Furthermore, the promising gas separation performances of hypoCOFs that we showed in Figure 4 suggest that the performance limits of experimentally synthesized COFs are still open to being upgraded.

### 3.2 COF/Polymer MMMs

**Figure 5:**
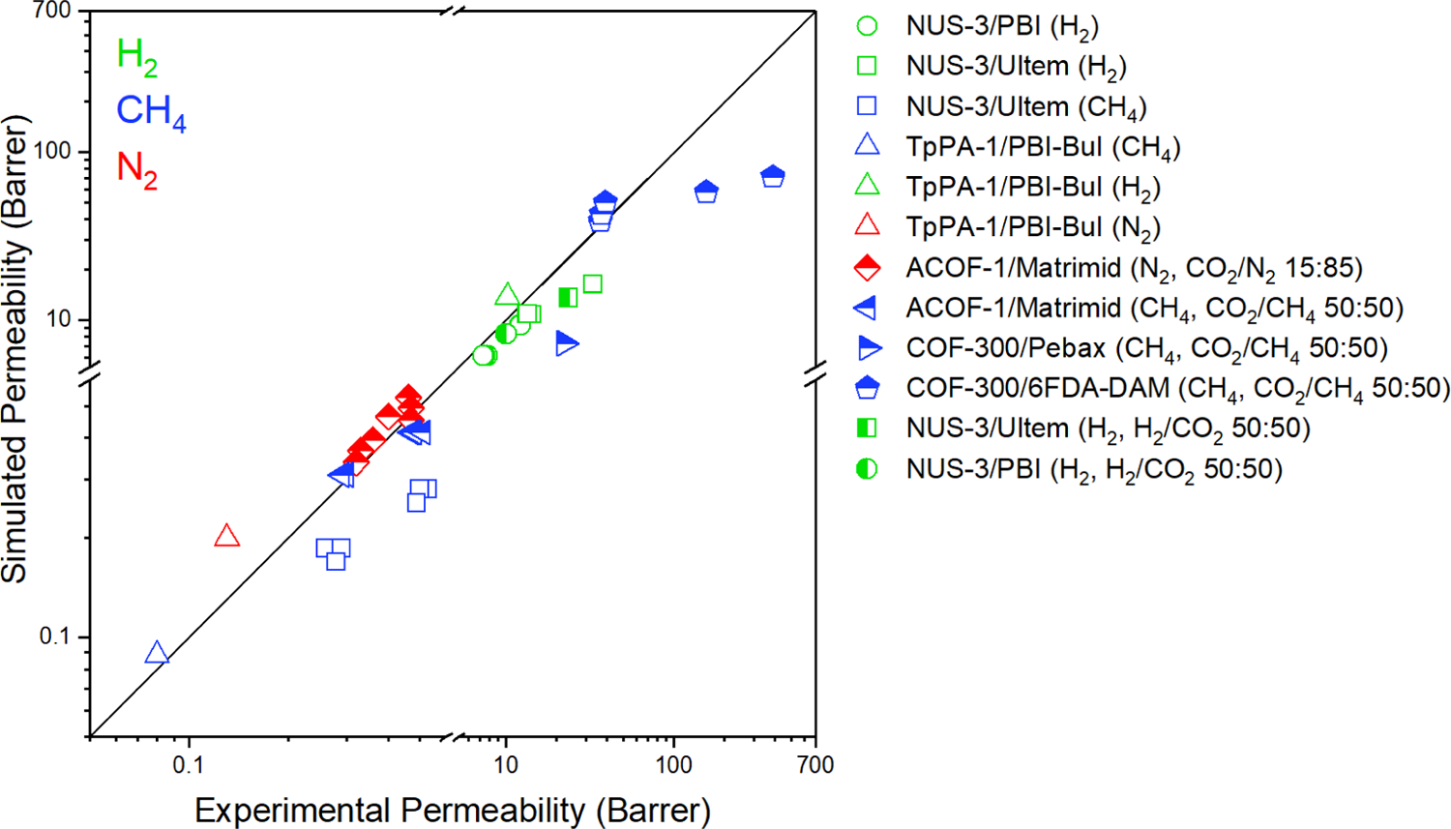
Comparison of experimental and computed gas permeabilities of COF/polymer MMMs. Green, red, and blue colors represent H_2_, N_2_, and CH_4_ permeabilities of MMMs, respectively. (Half-colored symbols show the mixture of related gases.)

We have so far shown that COFs and hypoCOFs are promising membranes due to their high gas permeabilities and selectivities; however, problems related to robustness, scalability, and mechanical stability are known to arise during the experimental manufacturing of membranes from crystal materials.^67^ Given that there are well-established fabrication techniques for polymeric membranes, using COFs as fillers in polymers to design MMMs can realize the high promises of COFs. Thus, we predicted permeabilities and selectivities of COF/polymer and hypoCOF/polymer MMMs made of different polymers for each gas separation considered in this work. To validate the accuracy of our computational approach for predicting gas separation performances of COF/polymer MMMs, we first calculated gas permeabilities of nine different COF/polymer MMMs reported in the literature under the same conditions as the experiments and compared the results in Figure 5. These MMMs were composed of four different COFs (NUS-3, ACOF-1, TpPA-1, and COF-300) and six different polymers (PBI, Ultem, Matrimid, PBI-BuI, Pebax, and 6FDA-DAM). Figure 5 shows the good agreement between experimental and computed permeability data of H_2_, N_2_, and CH_4_ (91 data points as listed in Table S7), indicating that our computational approach for calculating the gas permeabilities of COF-based MMMs is valid.

**Figure 6:**
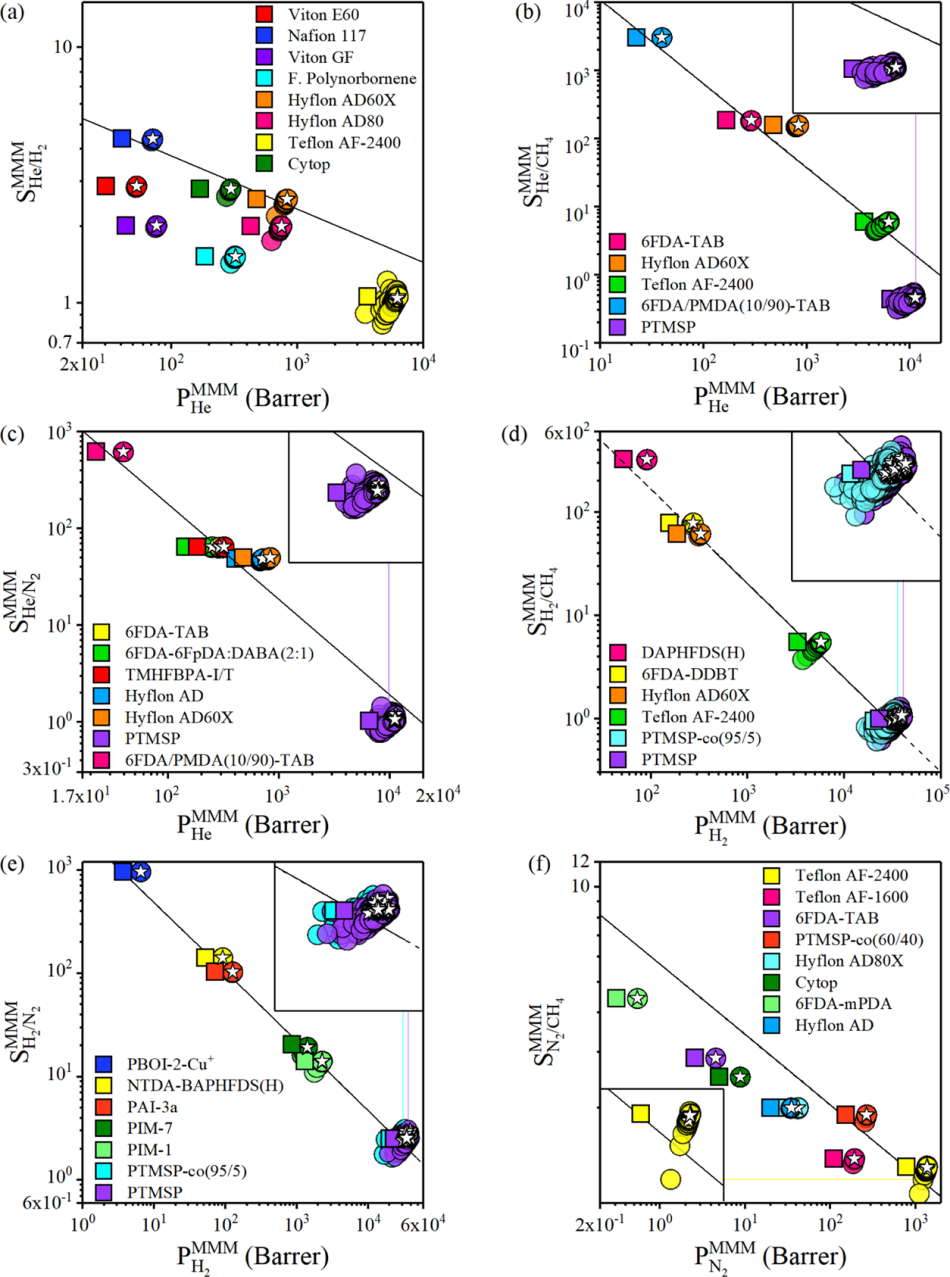
Computed permeabilities and selectivities of COF/polymer MMMs for (a) He/H_2_, (b) He/CH_4_, (c) He/N_2_, (d) H_2_/CH_4_, (e) H_2_/N_2_, and (f) N_2_/CH_4_ separations. Squares (circles) represent pure polymers (COF/polymer MMMs). HypoCOF/polymer MMMs are represented with white star-shaped data points. Robeson’s upper bound is shown as the black line.

In Figure 6, simulated gas permeabilities and selectivities were reported for the following COF/polymer MMMs: 4696 MMMs composed of 8 polymers and 587 COFs for He/H_2_ separation, 2940 MMMs composed of 5 polymers and 588 COFs for He/CH_4_ separation, 4116 MMMs composed of 7 polymers and 588 COFs for He/N_2_ separation, 3528 MMMs composed of 6 polymers and 588 COFs for H_2_/CH_4_ separation, 4116 MMMs composed of 7 polymers and 588 COFs for H_2_/N_2_ separation, and 4704 MMMs composed of 8 polymers and 588 COFs for N_2_/CH_4_ separation. For He/H_2_ separation (Figure 6a), the ranges of He permeabilities and selectivities of 4696 COF/polymer MMMs were computed to be between 52.5–6.28 × 10^3^ Barrer and 0.83-4.4, respectively. Except for Teflon AF-2400, the permeabilities of all polymers increased upon the incorporation of COF fillers since they have higher permeabilities than polymers. COF-based MMMs generated with Hyflon AD60X and Nafion-117 exceeded the upper bound due to the increase in He permeability.

Figure 6b shows that the upper limits of He permeability and He/CH_4_ selectivity are 1.13 × 10^4^ Barrer and 3.04 × 10^3^, respectively. Except for PTMSP, when COF fillers were incorporated into any of the polymers (6FDA-TAB, Hyflon AD60X, Teflon AF-2400, and 6FDA/PMDA(10/90)-TAB), He permeabilities increased up to 1.75 times and most of the MMMs surpassed the upper bound. Especially MMMs composed of 6FDA/PMDA(10/90)-TAB and Hyflon AD60X are the most promising ones since the increase in their He permeability is accompanied by high He/CH_4_ selectivities. As shown in Figure 6c, He permeability and He/N_2_ selectivity were computed to be between 38.9–1.13 × 10^4^ Barrer and 0.73–622, respectively. MMMs obtained with the incorporation of COFs into most of the polymers, such as 6FDA-TAB and TMHFBPA-I/T, outperformed the neat polymers in terms of permeability. As can be seen from Figure 6d for H_2_/CH_4_ separation, most of the COF-based MMMs are grouped around the upper bound. DAPHFDS(H) is the most promising polymer to generate MMMs since all COF-based MMMs consisting of this polymer were above the upper bound. For H_2_/N_2_ separation, as shown in Figure 6e, H_2_ permeability and selectivity vary in a wide range, 6.5–3.99 × 10^4^ Barrer and 1.6–960, respectively. All COF-based MMMs composed of PBOI-2-Cu^+^, NTDA–BAPHFDS(H), PAI-3a, and a large number of COF/PIM-7 and COF/PIM-1 MMMs surpass the upper bound. For N_2_/CH_4_ separation, as shown in Figure 6f, only the MMMs of PTMSP-co(60/40) and the majority of COF/Teflon AF-2400 MMMs could overcome Robeson’s upper bound.

Based on the results presented in Figure 6, we obtained three significant conclusions regardless of the gas separation type that we studied. (i) The identity of the COF determines the permeability of the MMM when the permeability of the polymer is high (>10^3^ Barrer). (ii) Once polymers exhibit low permeabilities and high selectivities, COF/polymer MMMs outperform the neat polymers generally only in terms of permeabilities. The gas permeability of MMM calculated with the Maxwell model at a volume fraction of 0.2 reduces to 1.75 times the gas permeability of the polymer when the gas permeability of COF is much higher than the gas permeability of the polymer.^68,69^ Therefore, selectivity, the ratio of gas permeabilities, does not change. (iii) Selectivities of polymers might improve, remain the same, or, in rare cases, diminish upon the incorporation of COF fillers if the polymers suffer from low selectivities.

We finally studied hypoCOF/polymer MMMs and showed the results in Figure 6 (stars). We studied 960 MMMs (8 polymers and 120 COFs) for He/H_2_, 600 MMMs (5 polymers and 120 COFs) for He/CH_4_, 840 MMMs (7 polymers and 120 COFs) for He/N_2_, 720 MMMs (6 polymers and 120 COFs) for H_2_/CH_4_, 840 MMMs (7 polymers and 120 COFs) for H_2_/N_2_, and 960 MMMs (8 polymers and 120 COFs) for N_2_/CH_4_ separation. Remarkably, hypoCOF/PTMSP, hypoCOF/PTMSP-co(95/5), and hypoCOF/Teflon AF-2400 MMMs show much higher permeabilities over neat polymers compared to the experimental COF/polymer MMMs. Incorporation of hypoCOFs into polymers boosted the He permeabilities of polymers for He/H_2_, He/N_2_, and He/CH_4_ separations, as shown in Figure 6a–c. All of the MMMs, including PTMSP and PTMSP-co(95/5), exceed the upper bound for H_2_/CH_4_ separation, and 111 of 120 hypoCOF/PTMSP MMMs and 113 of 120 hypoCOF/PTMSP-co(95/5) MMMs have higher H_2_/CH_4_ selectivities than the pure polymers, as shown in Figure 6d. MMMs of Teflon AF-2400, PTMSP, and PTMSP-co(95/5) with hypoCOFs have higher permeabilities than polymers. For H_2_/N_2_ separation, as shown in Figure 6e, all MMMs consisting of PTMSP and PTMSP-co(95/5) were above the upper bound, and the majority of the MMMs exhibited higher selectivities compared to pure polymers. N_2_ permeabilities of polymers were improved with the incorporation of hypoCOF fillers although selectivities remain the same, as shown in Figure 6f. Overall, the hypoCOFs that we selected based on the structural features of the high-performing CURATED COF membranes lead to the design of hypoCOF/polymer MMMs showing high gas permeabilities. This signals the importance of the targeted selection approach that we proposed to evaluate the promises of the enormous number of hypoCOFs both as membranes and as fillers in MMMs.

## 4 Conclusions

In this work, we used molecular simulations, GCMC and MD, to investigate the membrane-based gas separation potentials of experimental and hypothetical COFs for six different gas separations. He, H_2_, CH_4_, and N_2_ permeabilities and He/H_2_, He/CH_4_, He/N_2_, H_2_/CH_4_, H_2_/N_2_, and N_2_/CH_4_ selectivities of 589 COFs were computed, and the results showed that selectivities of COF membranes vary in the ranges of 0.12–3.5 for He/H_2_, 2.6 × 10^–2^–4.5 for He/CH_4_, 5.8 × 10^–2^–8.7 for He/N_2_, 4.1 × 10^–2^–6.3 for H_2_/CH_4_, 0.18–15.7 for H_2_/N_2_, and 0.13–3.2 for N_2_/CH_4_ separation. The surface area and porosity were identified as important structural properties to select the best-performing COFs exhibiting high gas permeabilities (>4 × 10^5^ Barrer). Based on the structure–permeability relation of the CURATED COFs that were identified to be above the upper bound for all six gas separations, 120 hypoCOFs were selected and examined as membranes. Results revealed that all of the selected hypoCOFs show high permeability and selectivity for each gas separation and exceed the upper bound except for He/H_2_ and N_2_/CH_4_ separations. Finally, 24 100 COF/polymer MMMs and 4920 hypoCOF/polymer MMMs were examined considering 25 different polymers, and results showed that permeability and selectivity of several polymers can be improved by the incorporation of COF fillers. The highest selectivities of COF-based MMMs were calculated as 4.4 for He/H_2_, 3041 for He/CH_4_, 622 for He/N_2_, 325 for H_2_/CH_4_, 960 for H_2_/N_2_, and 4.4 for N_2_/CH_4_ separation. In this work, we focused on a set of hypoCOFs, but there might be difficulties in the synthesis and applications of hypothetical materials. For example, hypoCOFs, which were identified to be the best membrane candidates, may not be easily synthesized, or the synthesized structures might have defects. Two other important points that will shape the future of COF membranes other than their separation performances are their stability and cost. The stability of COF membranes and COF-based MMMs needs to be tested under humid environments, in the presence of impurity gases, and at high temperature and pressure conditions. We believe that cost-related barriers for COF membranes will be overcome with the commercialization of COFs soon, provided that novel COF membrane processing methods will be discovered. We envisage that our work will inspire both experimental and computational studies for the investigation of COFs and hypoCOFs to be used in membrane-based gas separation applications.

## Supporting Information Available

Experimental gas permeabilities and membrane selectivities of neat polymers for He/H_2_, He/CH_4_, He/N_2_, H_2_/CH_4_, H_2_/N_2_, and N_2_/CH_4_ separations; experimental gas permeabilities and membrane selectivities of COF/polymer MMMs under different conditions; distribution of structural properties of all COFs and MOFs for each gas separation, and structure–gas permeability relations for all CURATED COFs and the ones that surpass the upper bound for all gas separations (PDF)
Properties of the best-performing MOF and COF membrane candidates, gas uptake, diffusivity, and permeability data for COFs (XLSX)
