# Supplementary material for: High-Throughput Screening of COF Membranes and COF/Polymer MMMs for Helium Separation and Hydrogen Purification
Source: ACS Appl Mater Interfaces. 2022 Apr 28;14(18):21738–49. doi: 10.1021/acsami.2c04016 (PMC9100491; doi:10.1021/acsami.2c04016)
Supplement: Supplementary file 1 — am2c04016_si_001.pdf [file am2c04016_si_001.pdf]

Supporting Information

for

High-throughput Screening of COF Membranes and COF/Polymer MMMs for Helium Separation and Hydrogen Purification

Sena Aydin[a], Cigdem Altintas[b], Seda Keskin[b*]

[a] Department of Computational Science and Engineering, Koc University, Rumelifeneri Yolu, Sariyer, 34450, Istanbul, Turkey

[b] Department of Chemical and Biological Engineering, Koc University, Rumelifeneri Yolu, Sariyer, 34450, Istanbul, Turkey

Submitted to *ACS Applied Materials & Interfaces*

[*]Corresponding author. Email: skeskin@ku.edu.tr Phone: +90(212)338 1362

Table S1: Experimental gas permeabilities and membrane selectivities of neat polymers for He/H_2 separation.

Polymers	P^{P}_{He} (Barrer)	S^{P}_{He/H_2}	Ref.
Viton E60 fluoroelastomer	30.5	2.87	1
Nafion 117	40.9	4.39	2
Viton GF fluoroelastomer	43.9	2.01	1
Cytop	170	2.8	3
Fluorinated Polynorbornene	185	1.52	4
Hyflon AD60X	476	2.55	5
Hyflon AD80	430	2	3
Teflon AF-2400	3600	1.06	6

Table S2: Experimental gas permeabilities and membrane selectivities of neat polymers for He/CH_4 separation.

Polymers	P^{P}_{He} (Barrer)	S^{P}_{He/CH_4}	Ref.
Polypyrrolone (6FDA/PMDA(10/90)-TAB)	22.5	3041	7
Polypyrrolone (6FDA-TAB)	166	184	7
Hyflon AD60X	476	157	5
Teflon AF-2400	3600	6	6
PTMSP	6500	0.433	8

Table S3: Experimental gas permeabilities and membrane selectivities of neat polymers for He/N_2 separation.

Polymers	P^{P}_{He} (Barrer)	S^{P}_{He/N_2}	Ref.
Polypyrrolone (6FDA/PMDA(10/90)-TAB)	22.5	622	7
Polypyrrolone (6FDA-TAB)	166	64.4	7
Polyimide (6FDA-6FpDA:DABA(2:1))	142	65	9
Polyarylate (TMHFBPA-I/T)	182	64.8	10
Hyflon AD	405	48.8	5
Hyflon AD60X	476	50.3	5
PTMSP	6500	1.03	8

Table S4: Experimental gas permeabilities and membrane selectivities of neat polymers for H_2/CH_4 separation.

Polymers	$P^{P}_{H_2}$ (Barrer)	$S^{P}_{H_2/CH_4}$	Ref.
Sulfonated Polyimide (DAPHFDS(H))	52	325	[11]
Polyimide (6FDA-DDBT)	156	78.8	[12]
Hyflon AD60X	187	61.7	[5]
Teflon AF-2400	3300	5.5	[6]
PTMSP-co(95/5)	20,400	0.953	[13]
PTMSP	23,200	0.995	[13]

Table S5: Experimental gas permeabilities and membrane selectivities of neat polymers for H_2/N_2 separation.

Polymers	$P^{P}_{H_2}$ (Barrer)	$S^{P}_{H_2/N_2}$	Ref.
Polybenzoxazinone imide (PBOI-2-Cu^{+})	3.7	960	[14]
Polyimide (NTDA-BAPHFDS(H))	52	141	[11]
Poly(amide-imide) (PAI)-3a	72	103	[15]
PIM-7	860	20.5	[16]
PIM-1	1300	14.1	[16]
PTMSP-co (95/5)	20,400	2.5	[13]
PTMSP	23,200	2.5	[13]

Table S6: Experimental gas permeabilities and membrane selectivities of neat polymers for N_2/CH_4 separation.

Polymers	$P^{P}_{N_2}$ (Barrer)	$S^{P}_{N_2/CH_4}$	Ref.
Polyimide (6FDA-mPDA)	0.31	4.43	[17]
Poly(pyrrolone)(6FDA-TAB)	2.58	2.87	[7]
Cytop	5	2.5	[3]
Hyflon AD80X	24	12	[3]
Hyflon AD	20	2	[3]
Teflon AF-1600	110	1.38	[3]
PTMSP-co(60/40)	153	1.9	[13]
Teflon AF-2400	790	1.3	[6]

Table S7: Experimental gas permeabilities and membrane selectivities of COF/polymer MMMs shown in Fig. 5 under different conditions.

COF Name	Polymer	Gas Molecules	P (bar)	T (K)	wt%	P^{MMM} (Barrer)	S^{MMM}	Ref.
NUS-3	PBI	CO_2	2	308	10	0.99	7.82 (H_2/CO_2)	18
		H_2				7.74		
		CO_2	3.5			0.89	8.42 (H_2/CO_2)	
		H_2				7.49		
		CO_2	5			0.86	8.51 (H_2/CO_2)	
		H_2				7.32		
		H_2	2		20	12.3	7.94 (H_2/CO_2)	
		CO_2				1.55		
		H_2	3.5			12.1	8.12 (H_2/CO_2)	
		CO_2				1.49		
		H_2	5			12.2	8.91 (H_2/CO_2)	
		CO_2				1.37		
	Ultem	H_2	2		10	14.5	2.46 (H_2/CO_2)	
		CH_4				0.26		
		H_2	3.5			13.3	2.3 (H_2/CO_2)	
		CH_4				0.29		
		CO_2				5.79	20 (CO_2/CH_4)	
		H_2	5			13.6	2.39 (H_2/CO_2)	
		CO_2				5.68		
		CH_4				0.28	20.3 (CO_2/CH_4)	
		H_2	2		20	33.4	2.23 (H_2/CO_2)	
		CO_2				15		
		CH_4				0.53	28.3 (CO_2/CH_4)	
		H_2	3.5			33.3	2.23 (H_2/CO_2)	
		CH_4				0.5		
		CO_2				15	30 (CO_2/CH_4)	
		H_2	5			33.2	2.39 (H_2/CO_2)	
		CH_4				0.49		
		CO_2				13.9	28.4 (CO_2/CH_4)	
	Ultem	CO_2	5		20	6.87	2.39 (H_2/CO_2)	
		H_2				23.89		
	PBI	CO_2			20	1.13	8.91 (H_2/CO_2)	
		H_2				10.04		
ACOF-1	Matrimid	CO_2	2		8	12.1	30.1 (CO_2/N_2)	19
		N_2				0.4		

		CO_2	3			11.4	31.6 (CO_2/N_2)	
		N_2				0.36		
		CO_2	4			11	32.8 (CO_2/N_2)	
		N_2				0.33		
		CO_2	5			10.4	32.9 (CO_2/N_2)	
		N_2				0.32		
		CO_2	2		16	17.7	34.7 (CO_2/N_2)	
		N_2				0.51		
		CO_2	3			16.5	36 (CO_2/N_2)	
		N_2				0.46		
		CO_2	4			15.9	34.2 (CO_2/N_2)	
		N_2				0.47		
		CO_2	5			15.5	33.9 (CO_2/N_2)	
		N_2				0.47		
TpPA-1	PBI-BuI	CH_4	20[a]		20	0.08	134.6 (H_2/CH_4)	20
		H_2				10.2	3.7 (H_2/CO_2)	
		N_2				0.13	78.4 (H_2/N_2)	
		CO_2				2.8	25.6 (CO_2/N_2)	
ACOF-1	Matrimid	CO_2	4		8	9.6	31.9 (CO_2/CH_4)	21
		CH_4				0.3		
		CO_2	5.5			9.1	31.8 (CO_2/CH_4)	
		CH_4				0.29		
		CO_2	7			8.9	31.2 (CO_2/CH_4)	
		CH_4				0.29		
		CO_2	8.5			8.9	30.5 (CO_2/CH_4)	
		CH_4				0.29		
		CO_2	10			8.8	30 (CO_2/CH_4)	
		CH_4				0.29		
		CO_2	4		16	15.3	32.4 (CO_2/CH_4)	
		CH_4				0.47		
		CO_2	5.5			14.9	31.2 (CO_2/CH_4)	
		CH_4				0.48		
		CO_2	7			14.7	29.1 (CO_2/CH_4)	
		CH_4				0.51		
		CO_2	8.5			14.5	28.3 (CO_2/CH_4)	
		CH_4				0.51		
COF-300	6FDA-DAM	CO_2	1	298	4[b]	842	23.5 (CO_2/CH_4)	22
		CH_4				36.5		
		CO_2			7.5[b]	972	26.2 (CO_2/CH_4)	
		CH_4				37.1		

		CO_2			13.2^b	1185	30.3 (CO_2/CH_4)	
		CH_4				39.2		
		CO_2			18.3^b	2842	24.6 (CO_2/CH_4)	
		CH_4				156.8		
		CO_2			26.2^b	4756	14.7 (CO_2/CH_4)	
		CH_4				390.5		
	Pebax	CO_2			3.4^b	81	19.4 (CO_2/CH_4)	
		CH_4				4.2		
		CO_2			8.3^b	86	21.5 (CO_2/CH_4)	
		CH_4				4		
		CO_2			11.5^b	98	32.7 (CO_2/CH_4)	
		CH_4				4.1		
		CO_2			16.1^b	107	25.5 (CO_2/CH_4)	
		CH_4				4.2		
		CO_2			23.3^b	327	14.7 (CO_2/CH_4)	
		CH_4				22.3		

a: atm, b: vol%.

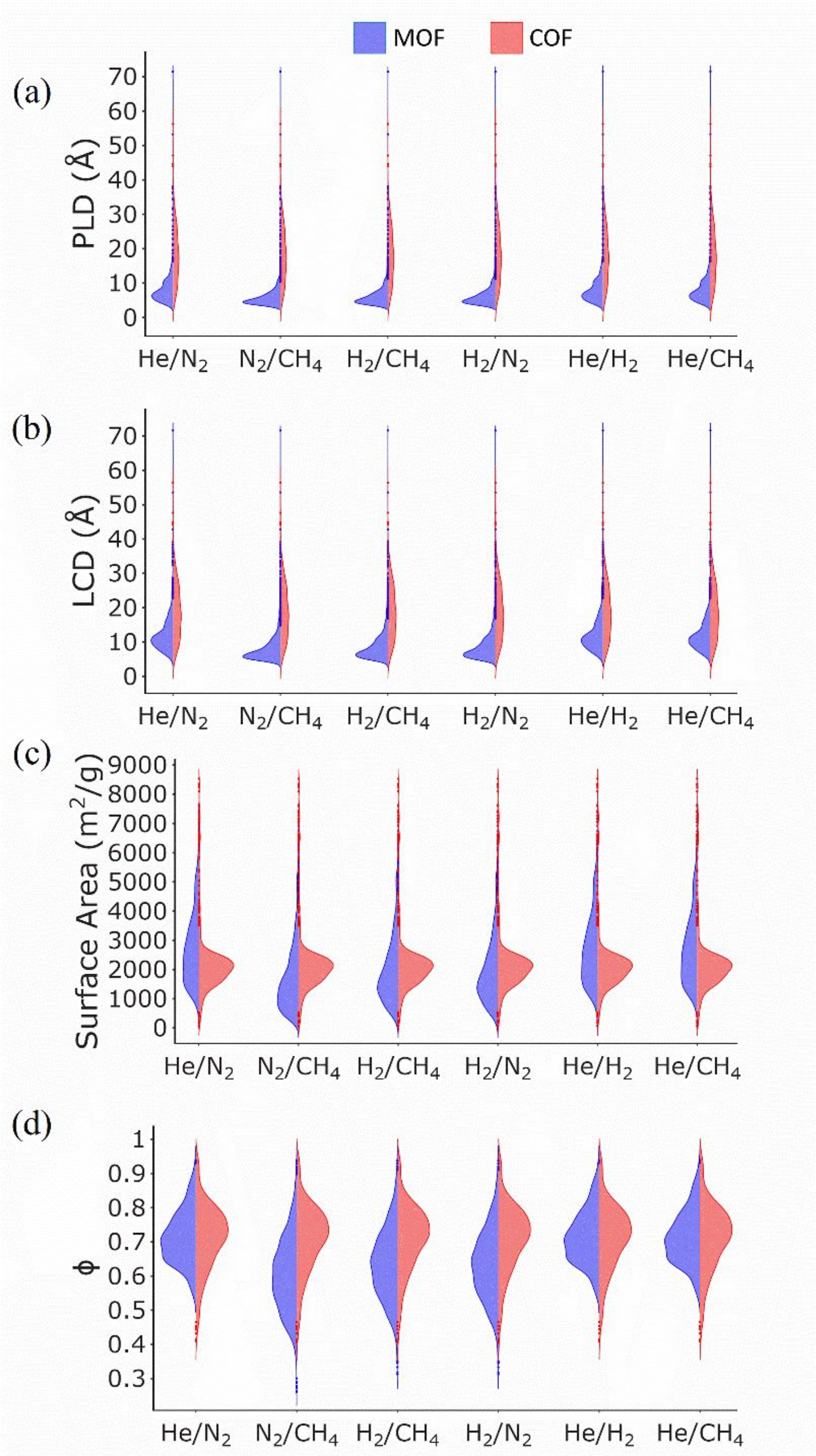

Figure S1. Distribution of (a) PLD, (b) LCD, (c) Sacc, (d) ϕ of all COFs and MOFs for each gas separation. Data for MOFs are taken from our previous study.[23]

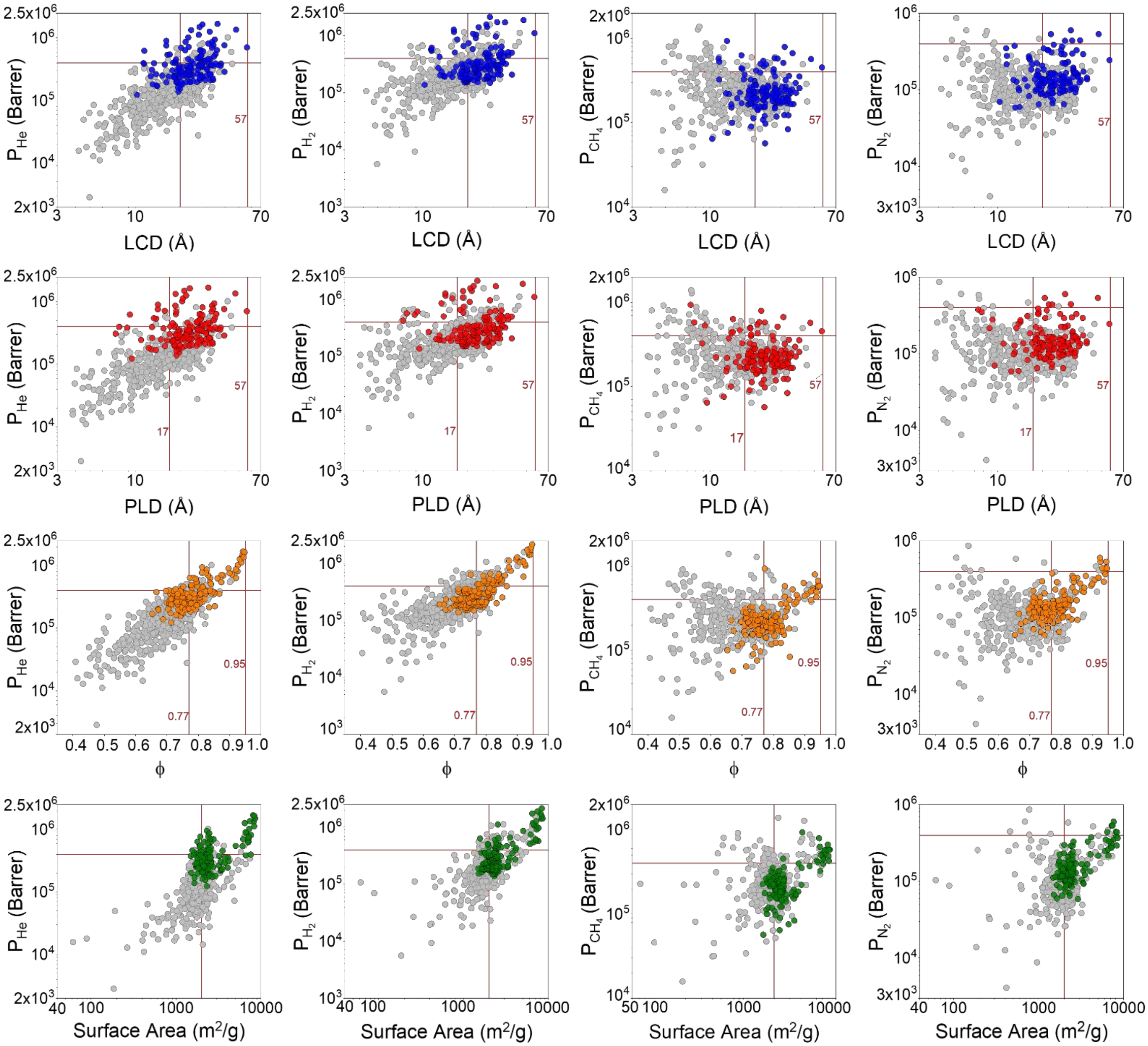

Figure S2. Structure-gas permeability relations for all 589 COFs (represented with gray data points) and 142 COFs which surpass the upper bound for all gas separations (represented with colored data points). Red lines show the specified structural property limits for 142 COFs with permeability >4×10^5 Barrer.

References

(1) Fitch, M.; Koros, W.; Nolen, R.; Carnes, J. Permeation of Several Gases through Elastomers, with Emphasis on the Deuterium/Hydrogen Pair. *J. Appl. Polym. Sci.* **1993**, *47* (6), 1033-1046.
(2) Chiou, J. S.; Paul, D. R. Gas Permeation in a Dry Nafion Membrane. *Ind. Eng. Chem. Res.* **1988**, *27* (11), 2161-2164.
(3) Merkel, T. C.; Pinnau, I.; Prabhakar, R.; Freeman, B. D. Gas and Vapor Transport Properties of Perfluoropolymers. In *Materials Science of Membranes for Gas and Vapor Separation*, Vol. 1; 2006.
(4) Teplyakov, V.; Paul, D.; Bespalova, N.; Finkel'Shtein, E. S. Gas Permeation in a Fluorine-Containing Polynorbornene. *Macromolecules* **1992**, *25* (16), 4218-4219.
(5) Macchione, M.; Jansen, J. C.; De Luca, G.; Tocci, E.; Longeri, M.; Drioli, E. Experimental Analysis and Simulation of the Gas Transport in Dense Hyflon AD60X Membranes: Influence of Residual Solvent. *Polymer* **2007**, *48* (9), 2619-2635.
(6) Pinnau, I.; Toy, L. G. Gas and Vapor Transport Properties of Amorphous Perfluorinated Copolymer Membranes Based on 2, 2-bistrifluoromethyl-4, 5-difluoro-1, 3-dioxole/tetrafluoroethylene. *J. Membr. Sci.* **1996**, *109* (1), 125-133.
(7) Zimmerman, C. M.; Koros, W. J. Polypyrrolones for Membrane Gas Separations. I. Structural Comparison of Gas Transport and Sorption Properties. *J. Polym. Sci. B Polym. Phys.* **1999**, *37* (12), 1235-1249.
(8) Toy, L.; Nagai, K.; Freeman, B.; Pinnau, I.; He, Z.; Masuda, T.; Teraguchi, M.; Yampolskii, Y. P. Pure-Gas and Vapor Permeation and Sorption Properties of Poly [1-phenyl-2-[P-(trimethylsilyl) phenyl] acetylene](PTMSDPA). *Macromolecules* **2000**, *33* (7), 2516-2524.
(9) Kim, J. H.; Koros, W. J.; Paul, D. R. Effects of CO_2 Exposure and Physical Aging on the Gas Permeability of Thin 6fda-Based Polyimide Membranes: Part 1. With Crosslinking. *J. Membr. Sci.* **2006**, *282* (1-2), 32-43.
(10) Guzmán-Gutierrez, M.; Ruiz-Trevino, F.; Zolutukhin, M.; Hernández-López, S.; Scherf, U. Gas Transport Properties of High Free Volume Polyarylates Based on Isophthalic/Terephthalic Acid Chloride Mixtures. *J. Membr. Sci.* **2007**, *305* (1-2), 347-352.
(11) Tanaka, K.; Islam, M. N.; Kido, M.; Kita, H.; Okamoto, K.-i. Gas Permeation and Separation Properties of Sulfonated Polyimide Membranes. *Polymer* **2006**, *47* (12), 4370-4377.
(12) Yang, L.; Fang, J.; Meichin, N.; Tanaka, K.; Kita, H.; Okamoto, K. Gas Permeation Properties of Thianthrene-5, 5, 10, 10-tetraoxide-Containing Polyimides. *Polymer* **2001**, *42* (5), 2021-2029.
(13) Nagai, K.; Higuchi, A.; Nakagawa, T. Gas Permeability and Stability of Poly(1-trimethylsilyl-1-propyne-co-1-phenyl-1-propyne) Membranes. *J. Polym. Sci. B: Polym. Phys.* **1995**, *33* (2), 289-298.
(14) Polotskaya, G.; Goikhman, M.; Podeshvo, I.; Kudryavtsev, V.; Pientka, Z.; Brozova, L.; Bleha, M. Gas Transport Properties of Polybenzoxazinoneimides and Their Prepolymers. *Polymer* **2005**, *46* (11), 3730-3736.
(15) Fritsch, D.; Avella, N. Highly Gas Permeable Poly (Amide Imide)S. In *36th IUPAC International Symposium on Macromolecules, August*, 1996; pp 4-9.
(16) Budd, P. M.; Msayib, K. J.; Tattershall, C. E.; Ghanem, B. S.; Reynolds, K. J.; McKeown, N. B.; Fritsch, D. Gas Separation Membranes from Polymers of Intrinsic Microporosity. *J. Membr. Sci.* **2005**, *251* (1-2), 263-269.
(17) Wang, L.; Cao, Y.; Zhou, M.; Ding, X.; Liu, Q.; Yuan, Q. The Gas Permeation Properties of 6FDA-2, 4, 6-Trimethyl-1, 3-Phenylenediamine (TMPDA)/1, 3-Phenylenediamine (mPDA) Copolyimides. *Polym. Bull.* **2008**, *60* (1), 137-147.
(18) Kang, Z.; Peng, Y.; Qian, Y.; Yuan, D.; Addicoat, M. A.; Heine, T.; Hu, Z.; Tee, L.; Guo, Z.; Zhao, D. Mixed Matrix Membranes (MMMs) Comprising Exfoliated 2D Covalent Organic Frameworks (COFs) for Efficient CO_2 Separation. *Chem. Mater.* **2016**, *28* (5), 1277-1285.
(19) Shan, M.; Seoane, B.; Andres-Garcia, E.; Kapteijn, F.; Gascon, J. Mixed-Matrix Membranes Containing an Azine-Linked Covalent Organic Framework: Influence of the Polymeric Matrix on Post-Combustion CO_2-Capture. *J. Membr. Sci.* **2018**, *549*, 377-384.
(20) Biswal, B. P.; Chaudhari, H. D.; Banerjee, R.; Kharul, U. K. Chemically Stable Covalent Organic Framework (COF)-Polybenzimidazole Hybrid Membranes: Enhanced Gas Separation through Pore Modulation. *Chem. Eur. J.* **2016**, *22* (14), 4695-4699.
(21) Shan, M.; Seoane, B.; Rozhko, E.; Dikhtiarenko, A.; Clet, G.; Kapteijn, F.; Gascon, J. Azine-Linked Covalent Organic Framework (COF)-Based Mixed-Matrix Membranes for CO_2/CH_4 Separation. *Chem. Eur. J.* **2016**, *22* (41), 14467-14470.

(22) Cheng, Y.; Zhai, L.; Ying, Y.; Wang, Y.; Liu, G.; Dong, J.; Ng, D. Z. L.; Khan, S. A.; Zhao, D. Highly Efficient CO_2 Capture by Mixed Matrix Membranes Containing Three-Dimensional Covalent Organic Framework Fillers. *J. Mater. Chem. A* **2019**, *7* (9), 4549-4560.
(23) Daglar, H.; Aydin, S.; Keskin, S. MOF-Based MMMs Breaking the Upper Bounds of Polymers for a Large Variety of Gas Separations. *Separation and Purification Technology* **2022**, *281*, 119811.
